# Outcomes of Children Born to Pregnant Women With Drug-resistant Tuberculosis Treated With Novel Drugs in Khayelitsha, South Africa: A Report of Five Patients

**DOI:** 10.1097/INF.0000000000003069

**Published:** 2021-02-02

**Authors:** Rebecca Acquah, Erika Mohr-Holland, Johnny Daniels, Jennifer Furin, Marian Loveday, Vanessa Mudaly, Anja Reuter

**Affiliations:** From the * Médecins Sans Frontières, Khayelitsha, Cape Town, South Africa; † Department of Global Health and Social Medicine, Harvard Medical School, Boston, MA; ‡ Health Systems Research Unit, South African Medical Research Council, KwaZulu-Natal, South Africa; § Department of Health, Province of the Western Cape, Cape Town, South Africa.

**Keywords:** tuberculosis, pregnancy, bedaquiline, linezolid, delamanid

## Abstract

This brief report presents a series of 5 pregnant women treated for rifampicin-resistant tuberculosis with the novel drugs bedaquiline, delamanid, and linezolid as part of an optimized backbone regimen and reviews the outcomes of the children born to them. Although the case series is small, all children had excellent birth outcomes suggesting pregnant women should not be denied access to novel therapies for RR-TB.

Tuberculosis (TB) is a leading cause of maternal mortality, and pregnant women are a vulnerable population when it comes to all forms of TB, including drug-resistant (DR) disease.^1^ While there are limited data on the number of pregnant women who develop DR-TB each year, morbidity and mortality is common in this group.^2^ Studies have shown that pregnant women with DR-TB may be offered substandard care, face a high degree of discrimination both from health care workers and from others in their communities, and are systematically denied access to treatment innovations that have been associated with decreased morbidity and mortality in other populations.^3^

Not only do these issues affect the health of the pregnant women but also the lives of their unborn children. Children born to pregnant women with TB are at increased risk of prematurity, hospitalization, and death.^4,5^ One study of 10 pregnant women with DR-TB in Ukraine found that women with DR-TB had a higher rate of pregnancy complications than pregnant women who did not have DR-TB. They also experienced higher rates of treatment-associated adverse events than nonpregnant women with DR-TB, and these adverse events could have affected their unborn babies.^6^ In addition to the challenges faced during pregnancy and DR-TB treatment, conflicting advice about optimal postpartum practices in these women—including separation of mother-infant pairs and whether or not to breastfeed—can put neonates at risk.^7^

New and repurposed TB medications such as bedaquiline, linezolid, and delamanid, stand to improve the lives and health of pregnant women with DR-TB and their unborn children for several reasons.^8^ First, their use is associated with more rapid time to sputum conversion—and thus decreased infectiousness, better treatment outcomes, and decreased mortality. Second, these medications appear to have a better safety profile than some of the medications more commonly used in treatment of DR-TB such as the aminoglycosides—which are known to cause both maternal and fetal ototoxicity—and ethionamide, which has been associated with severe vomiting, hypothyroidism and the development of neural tube defects. Because pregnant women are systematically excluded from most drug trials,^9^ there are limited data on the use of these newer medications during pregnancy and their effects on the children born to such women. Recent data from a cohort of women in South Africa suggest bedaquiline can be given safely to pregnant women, although there was a higher risk of the babies being born with low birthweight among the women receiving this medication.^10^ More information, however, is needed on the long-term effects on children born to women who received new and repurposed TB medications during pregnancy. Here, we report on the clinical characteristics, treatment outcomes, and offspring born to 5 women who received bedaquiline, delamanid, and linezolid for DR-TB treatment while pregnant.

## METHODS

We conducted a retrospective record review of a cohort of patients receiving treatment for DR-TB in Khayelitsha, Cape Town, South Africa, over a 5-year time period beginning in 2014. A total of 1127 patients were treated during this time period, and from this cohort, 5 pregnant women were identified who received either bedaquiline, delamanid, or linezolid during treatment for their DR-TB. Of note, although all female DR-TB patients of child-bearing age undergo routine pregnancy testing before treatment initiation and are offered free contraceptive services throughout treatment, routine monthly pregnancy testing was not performed in this cohort, so we are not able to report on women who may have had early pregnancies that resulted in abortion or miscarriage. These women were assessed on a monthly basis throughout their pregnancies, and their children were followed up routinely as recommended by the National Department of Health of South Africa. Adverse events in women were documented in routine clinical files, and serious adverse events were defined as those (1) resulting in death; (2) that are life-threatening; (3) that require inpatient hospitalization or prolongation of existing hospitalization; (4) that resulted in persistent or significant disability/incapacity; or (5) those which resulted in congenital anomaly/birth defect. For the DR-TB treatment outcomes, standard WHO definitions were used. Children were followed for different time periods ranging from age 2 months to 24 months. For children’s outcomes, they were considered well if they were within their weight for age bands and if they met all developmental milestones defined for their age groups. This case series was approved by the ethics review board at Medecins Sans Frontieres and the Human Research Ethics Committee at the University of Cape Town, South Africa (HREC 499/2011).

## RESULTS

The age range of the women was between 18 and 29 years. Table 1 reviews the clinical characteristics, drug exposure, and outcomes of both the mother and the infant.

**TABLE 1.** Clinical Characteristics, Drug Exposures, and Outcomes Among Women and Children Where New TB Drugs Were Used During Pregnancy

	Patient 1	Patient 2	Patient 3	Patient 4	Patient 5
HIV status	Positive	Negative	Positive	Positive	Negative
ART status	Naive	–	TDF/FTC/EFV	Lost to follow-up	–
CD4 count	84	–	82	94	–
Viral load	–	–	<40 copies/mL	243,827 copies/mL	–
TB resistance profile	INH and RIF	INH and RIF	INH and RIF	RIF	RIF
Indication for new or repurposed TB drugs	Renal impairment, salvage regimen	Substitute for injectable	Severe disease	Standard of care	Substitute for injectable, salvage regimen
Drug exposure (d of pregnancy)	Delamanid (310) Bedaquiline (104)	Linezolid (154)	Linezolid (46)	Linezolid (21) Bedaquiline (21)	Delamanid (9) Linezolid (61) Bedaquiline (138)
Trimester exposure	1–3	1–3	3	3	2–3
Pregnancy outcome	Delivery at term	Delivery at term	Delivery at term	Delivery at term	Delivery at term
Breast-feeding?	No	Unknown	No	Yes	No
Maternal TB outcome	Cured	Lost to follow-up (mo 18)	Cured	Lost to follow-up (mo 4)	Cured
Neonatal outcomes (with month of last follow-up visit in parenthesis)	Well (18)	Well (12)	Well (8)	Well (2)	Well (24)

ART indicates antiretroviral; HIV, human immunodeficiency virus; INH, isoniazid; RIF, rifampicin.

The medications delamanid, bedaquiline, and linezolid appeared to have been well-tolerated by the pregnant women, and there were no serious adverse events reported in the women during their pregnancies or in the postpartum period. Three of the 5 women also received levofloxacin. Although 2 of the mothers did not have successful DR-TB treatment outcomes and were given an outcome of “lost-to-follow-up,” their children were all considered to be doing well at the time of the final assessment, conducted between 2 and 24 months postpartum. None of the babies were premature, all were live-born, and none developed TB or DR-TB. None of the babies had congenital abnormalities. One baby was breast-fed, 3 were formula fed, and the feeding method of 1 baby was not recorded in the chart.

## DISCUSSION

Although this cohort of pregnant women is small, the outcomes of the children are encouraging: all 5 of them were born alive and were growing and developing normally at their follow-up visits. More information is clearly needed, but these results support the idea that newer and repurposed TB medications can and should be safely given to women when they are pregnant.

There were several concerning findings in this population, however. Although 3 of the 5 pregnant women (60%) had successful treatment outcomes—a success rate on par with that seen in the general population being treated for DR-TB globally—the other 2 women were unable to successfully complete their prescribed therapy and were assigned a “lost-to-follow-up” treatment outcome. More work is needed to determine the unique challenges that women with DR-TB may face during treatment and to tailor interventions to address them. It is also worth mentioning that only 1 of the women was able to breastfeed her child. Although a more detailed exploration of the reasons the women gave for not choosing to breastfeed would have added to the data in the study, clearly there is a need to better promote optimal feeding for the children born to women with DR-TB.

## CONCLUSION

In this case series of pregnant women exposed to the novel and repurposed medications bedaquiline, delamanid, and linezolid used in the treatment of DR-TB, treatment outcomes were comparable to other populations, and babies born to these women were all full-term who did not experience any developmental abnormalities. Although more data is needed, this case series, taken in the context of the limited literature that has been published, shows that there is no justification for denying pregnant women living with DR-TB access to life-saving and innovative therapeutic regimens containing bedaquiline, delamanid, and linezolid. Careful consideration and inclusion of pregnant women in clinical trials involving these medications would provide a clearer evidence base. Because pregnant women are systematically excluded from such clinical trials, however, most antituberculous drugs are approved with an undetermined risk in pregnancy, and it can be decades before such safety data are collected and analyzed. This knowledge gap could also be supplemented by the development and use of DR-TB pregnancy registers, which have been successful in large HIV cohorts and which would provide much needed programmatic data.
